# Coexistence of Satellite Ground Stations in Teleport Facilities: Interference Assessment, Real Application Scenario and Measurements

**DOI:** 10.3390/s22031234

**Published:** 2022-02-06

**Authors:** Nektarios Moraitis, Athanasios D. Panagopoulos

**Affiliations:** School of Electrical and Computer Engineering, National Technical University of Athens, 9 Heroon Polytechniou Str., Zografou, 15773 Athens, Greece; morai@mobile.ntua.gr

**Keywords:** broadcast satellite service (BSS), carrier-to-interference ratio (CIR), fixed satellite service (FSS), interference satellite terminals

## Abstract

This article introduces a unified and detailed methodology for interference assessment between coexisting fixed satellite service (FSS) and broadcast satellite service (BSS) with spectrum sharing at the Ka-band. The interference analysis is presented along with a step-by-step algorithm for the calculation of the carrier-to-interference ratio (CIR). The proposed procedure takes into consideration the near-field effect of ground-satellite-terminal antennas since these may reside at very close distances. Furthermore, numerical results are delivered so as to assess the CIR in relation to the relative geometry and the technical characteristics of the satellite terminals. A real application scenario is also provided along with measurements so as to validate the recommended methodology. Finally, mitigation techniques are proposed for the protection of the victim stations and operation under harmful interference conditions.

## 1. Introduction

Satellite networks play a vital role in Internet connectivity on a global scale. There has been a global expansion of satellite services during the few last years with the establishment of many satellite terminals and new satellite teleports by stakeholders [1]. This trend is directly related to the increasing demand of communication services, and the high-throughput satellite experience with high efficiency for the consumers. The advent and the development of fifth-generation (5G) networks are expected to boost the growth of satellite service needs in the upcoming future. Due to the inherent nature of the satellite systems to provide coverage in wide areas and especially to remote areas, their integration with 5G will support various scenarios, such as enhanced mobile broadband, massive machine-type communications, and ultra-reliable and low-latency communications [2].

This enormous increase of bandwidth-demanding services forced the satellite providers to improve space-segment performance, enhancing the link capacity. New spectrum parts (e.g., Ku, Ka, and Q/V frequency bands) have been devoted to satellite communications. In this context, for the fixed satellite service (FSS), an exclusive spectrum has been allocated, but there are also spectrum parts that are being shared between FSS and other radio services, such as fixed service (FS) or feeder links for the broadcasting satellite service (BSS) [3]. In the United States, the domestic implementation of the internationally known BSS is called the direct broadcast satellite service (DBS) [4]. The deployment of many new ground stations with spectrum sharing, especially in the 17.3–20.2 GHz segment, entails the risk of harmful interference, where the uplink segment of a BSS feeder link may interfere with the downlink segment of a FSS [3]. Therefore, upon the establishment of new satellite terminals (e.g., in teleports) with spectrum sharing in the Ka-band, it is of high priority to assess the potential ground-path interference and, in the case of occurrence, propose any available mitigation countermeasures. The interference assessment between satellite terminals and FS or 5G networks has already been investigated in [5,6,7,8], also taking into account the impact of tropospheric propagation. Furthermore, International Telecommunications Union Radio (ITU-R) has introduced methods for the determination of the coordination area around any type of earth station that shares a spectrum with terrestrial radiocommunications services when more than one satellite provider is involved [9]. However, in the case of spectrum sharing between FSS and BSS within a teleport administration at very close distances, these methods are not applicable.

The purpose of this contribution is to assess accurately the ground-path interference caused between ground satellite–ground stations (gateways), operating at the Ka-band, which are collocated in teleport facilities. The scenario involves the downlink part of an FSS terminal to be interfered with by the uplink of one or more adjacent BSS feeder-link stations. A detailed methodology is introduced that takes into consideration the relative geometry of the stations, the propagation environment, and the technical characteristics of the satellite terminals. Furthermore, special care is taken for the antenna gains since it is foreseen that the terminals are established at close distances and within the near field of each other. To the authors’ knowledge, it is the first time that a thorough and straightforward procedure is provided for the interference calculation between ground satellite stations, considering the near-field effect of the antennas as well. The interference is evaluated for one or multiple interferers (total interference) in terms of carrier-to-interference ratio (CIR) at the victim station.

Section 2 describes analytically the interference scenario and the analysis along with the proposed algorithmic procedure for its calculations. Numerical results are also provided, assessing the impact of various parameters (e.g., elevation and azimuth angle, distance, relative position, and number of interferers) of the ground stations to the obtained CIR. In Section 3, the recommended methodology is applied in a real scenario, quantifying the possible interference in a small teleport facility, installing new three BSS stations sharing a frequency spectrum with a FSS gateway. The proposed procedure is validated by interference measurements in the same teleport facility, presented in Section 4. Finally, Section 5 describes the application of different techniques for interference mitigation, followed by interesting conclusions in Section 6.

## 2. Interference Scenario and Analysis

### 2.1. Interference Calculation Methodology

**Figure 1:**
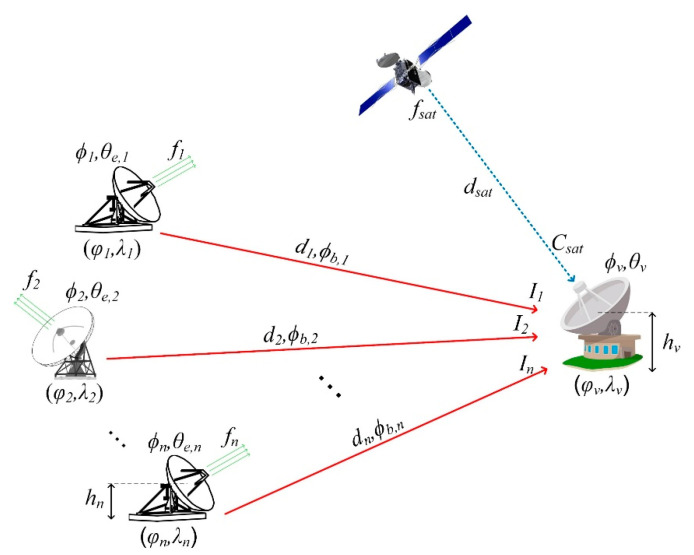
Ground-path interference scenario.

The scenario comprises the coexistence of multiple satellite earth stations that could potentially cause interference to nearby ground stations. This issue is commonly encountered in satellite antenna parks (e.g., teleport facilities) where many ground stations coexist at distances that do not exceed 1 km. This entails that the interference analysis should take into consideration that almost all the ground station antennas (interferers and victim) reside in the near field of one another. Interference may occur when new ground stations are installed in the vicinity of an existing antenna, and their transmission frequencies coincide with the reception-frequency part of the victim antenna. (The opposite may occur as well.) The examined scenario is depicted in Figure 1, considering the interference caused from *n* ground stations (*n* = 1 … N), to an existing, single satellite terminal (victim). If other victims exist, the following methodology is applied, independently, for each one of them. In this case, the transmission frequency *f_n_* of each interfering station (uplink) overlaps with the reception frequency *f_sat_* of the victim (downlink). Furthermore, *ϕ_n_* and *θ_e,n_* indicate the azimuth and elevation angles, respectively, in degrees, to which each *n* interferer’s antenna dish is oriented. Finally, *ϕ_v_* and *θ_v_* are the azimuth and elevation angles, in degrees, of the victim’s antenna.

The first step is to calculate the power of the carrier signal that arrives from the satellite to the victim station, which is determined using the fundamental transmission theory [10]

(1) $$C_{sat}=EIRP_{sat}+G_{Rx,\max}-L_{v}^{Rx}-20\log_{10}(f_{sat})-20\log_{10}(d_{sat})-92.45,$$

where *C_sat_* (in dBW) is the carrier power received at the victim terrestrial station from the satellite; *EIRP_sat_* denotes the equivalent isotropic radiated power (in dBW) transmitted by the corresponding satellite; *G_Rx,max_* stands for the maximum gain (in dBi) of the receiving ground antenna; *f_sat_* is the reception frequency (in GHz); *d_sat_* represents the distance of the earth-space link, in kilometers, between the victim station and the orbiting satellite; and $L_{v}^{Rx}$ indicates any equipment losses at the victim ground station, in decibels. The constant parameter in Equation (1) is yielded from the free-space loss equation [10], so as to represent the frequency in GHz and the distance in kilometers. If *G_Rx,max_* is unknown, the gain, directly in dBi, can be determined by exploiting the antenna’s dish diameter, according to

(2) $$G_{Rx,max}=20.4+10\log_{10}(k)+20\log_{10}(d_{ant})+20\log_{10}(f_{sat}),$$

where *k* is the efficiency factor (*k* = 0.55–0.6), *f_sat_* is the reception frequency (in GHz), and *d_ant_* the dish diameter of the antenna in meters [11]. The direct distance *d_sat_* between the satellite and the victim can be calculated according to

(3) $$d_{sat}=\sqrt{r_{d}^{2}+R_{E}^{2}-2R_{E}r_{d}\sin\left[\theta_{v}+\sin^{-1}\left(\frac{R_{E}}{r_{d}}\cos\theta_{v}\right)\right]},$$

where *r_d_* denotes the geostationary orbit radius (*r_d_* = 42,164 km from the center of the earth), *R_E_* represents the mean Earth radius (6378 km), and *θ_v_* stands for the elevation angle of the victim station [12].

The second step is to calculate the ground distance *d_n_* between each interferer and the victim, along with the bearing angle *ϕ_b,n_*, as shown in Figure 1. The bearing angle is considered by counting clockwise with respect to True North, with its direction from the interferer to the victim. The location coordinates of each interfering station are *φ_n_*, *λ_n_*, whereas the victim coordinates are *φ_v_*, *λ_v_*, designating the latitude and longitude, respectively. The coordinate parameters are usually provided in decimal degrees. Applying the Havershine formula [13], the distance, in meters, and the bearing angle, in degrees, can be determined by

(4) $$d_{n}=2R_{E}\sin^{-1}\left(\sqrt{\sin^{2}\left(\frac{\Delta \varphi }{2}\right)+\cos\varphi_{n}\cos\varphi_{v}\sin^{2}\left(\frac{\Delta \lambda }{2}\right)}\right),$$

(5) $$\phi_{b,n}=\frac{180}{\pi }\tan^{-1}\left(\frac{\sin(\Delta \lambda )cos\varphi_{v}}{cos\varphi_{n}\sin\varphi_{v}-\sin\varphi_{n}cos\varphi_{v}\cos(\Delta \lambda )}\right),$$

where *R_E_* is the mean Earth radius, *φ_n_* denotes the latitude of each interferer, and *φ_v_* indicates the latitude of the victim station. Furthermore, Δ*φ* = *φ_v_*–*φ_n_* and Δ*λ* = *λ_v_*–*λ_n_* are the latitude and longitude phase differences. The decimal degrees of the coordinates should be converted in radians before applying Equations (4) and (5). It should be pointed out that the ground-path distance *d_n_* and the direct distance (in three dimensions) between the feeders of each antenna (i.e., at the center of the dishes), are similar since *d_n_* >> {*h_n_*, *h_v_*}, where *h_n_*, *h_v_* are the effective heights of the *n*-th interfering ground station and the victim station, respectively. The effective height is considered as the height of the dish center in meters above the ground, as shown in Figure 1.

The third step is to calculate the antenna off bore-sight gains between each *n* interfering and the interfered station, based on their relative azimuth and elevation angles. For this purpose, the off bore-sight angle has to be determined, which is provided by

**Figure 2:**
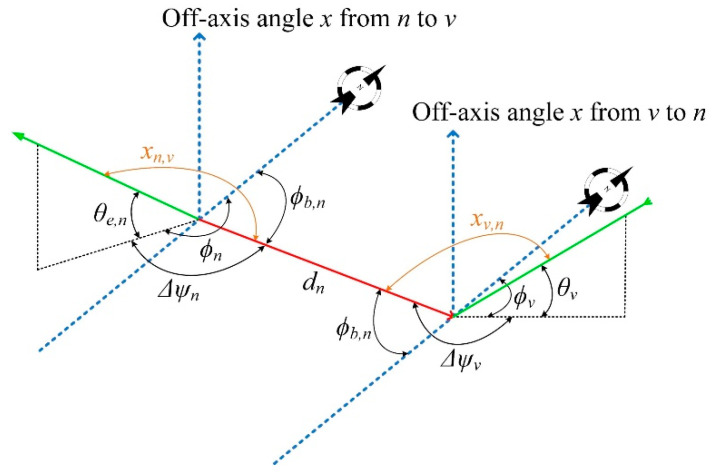
Three-dimensional geometry to define the off bore-sight angle between an interfering and victim station.

(6) cosx=cosΔψcosθ,

where *x* is the off bore-sight angle, Δ*ψ* stands for the angle in the horizontal plane between the line from *n*-th station to the victim station (ground-path *d_n_*) and the ground projection of the slant path, and *θ* is the elevation angle of the antenna [5]. The three-dimensional geometry for the aforementioned angles is shown in Figure 2, where one can determine the off bore-sight angle of the *n*-th transmitter in the direction of the receiver, and the off bore-sight angle of the victim’s receiver in the direction of each *n*-th transmitter.

Based on Figure 2 and after a few trigonometric calculations, Δ*ψ* can be expressed as a function of the bearing angle and the azimuth of each station. Therefore, Δ*ψ_n_* = *ϕ_n_*–*ϕ_b,n_*, and Δ*ψ_v_* = *π* + *ϕ_b,n_*–*ϕ_v_*, so the off bore-sight angles from (6) can be expressed as

(7) $$x_{n,v}=\cos^{-1}\left(\cos\left(\phi_{n}-\phi_{b,n}\right)\cos\theta_{e,n}\right),$$

(8) $$x_{v,n}=\cos^{-1}\left(\cos\left(\pi +\phi_{b,n}-\phi_{v}\right)\cos\theta_{v}\right).$$

**Figure 3:**
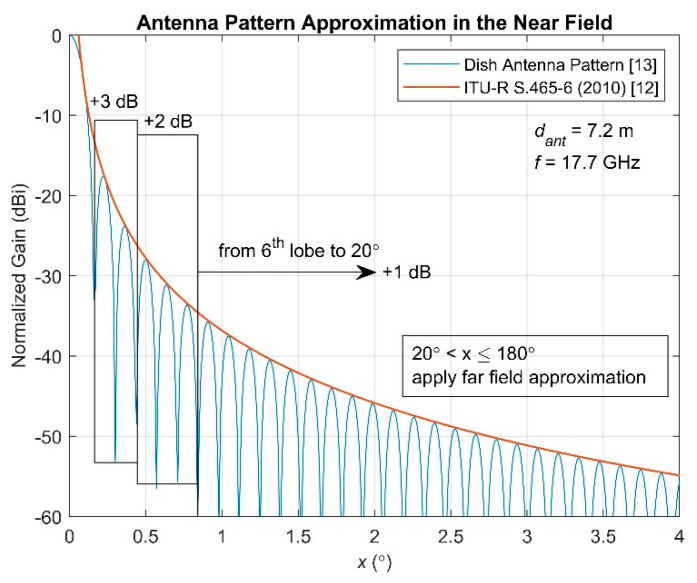
Antennae gain approximations with respect to the off bore-sight angle, taking into consideration the near field.

From the off bore-sight angle, the off bore-sight gains at both sides of the link can be calculated based on the antenna patterns. If the antenna patterns are not available, or the provided information is inadequate to determine the exact gain, the antenna gains at the specific angles can be calculated according to the simplified following expression:

(9) $$G(x)=\left\{ \begin{array}{l} 32-25\log_{10}(x),x\leq 48^{\circ }\\ -10,{\;48}^{\circ }<x\leq 180^{\circ } \end{array}\right.,$$

where *G*(*x*) is the gain (in dBi) and *x* is the off bore-sight angle in degrees [14], which stands in the far-field region of the antenna. Furthermore, the gain of a dish antenna can be described by

(10) $$G(x)=20\log_{10}\left(2\frac{J_{1}\left(\frac{\pi d_{ant}\sin(x)}{\lambda }\right)}{\frac{\pi d_{ant}\sin(x)}{\lambda }}\right),$$

where *G*(*x*) is the gain (in dBi), *x* is the off bore-sight angle in radians, *d_ant_* denotes the diameter of aperture in meters, *λ* is the signal wavelength in meters, and *J_1_*(∙) represents the Bessel function of the first kind [15]. A graphical representation of Equations (9) and (10) is provided in Figure 3 for the first few degrees of *x*. The results in (10) are obtained for *d_ant_* = 7.2 m and *f* = 17.7 GHz. One can observe that the envelope of Equation (10) is approximated by Equation (9). According to [16,17], in the near field of any antenna, the nulls of the lobes are not still formed, and the first lobes of the pattern are increased by a constant factor in decibels, as is also depicted in Figure 3. More specifically, a valid assumption is that the first two lobes, after the main lobe, are increased by about 3 dB, the next three lobes are increased by about 2 dB, and from the sixth lobe up to 20°, the gain is increased by 1 dB. Finally, after 20°, the gain is approximated by the far-field expression based on [14].

According to the above, in the proposed methodology, the near-field antenna gain is approximated exploiting the following closed form expression:

(11) $$G(x)=\left\{ \begin{array}{l} 32-25\log_{10}(x)+3,\;0.16^{\circ }\leq x\leq 0.44^{\circ }\\ 32-25\log_{10}(x)+2,\;0.44^{\circ }<x\leq 0.85^{\circ }\\ 32-25\log_{10}(x)+1,\;0.85^{\circ }<x\leq 20^{\circ }\\ 32-25\log_{10}(x),{\;20}^{\circ }<x\leq 180^{\circ } \end{array}\right..$$

The next step is to calculate the received interference at the victim station, individually, for each *n*-th interfering station. The interference power received by the victim station *v*, from ground station *n*, is calculated by

(12) $$I_{n}=P_{n}^{Tx}+G_{n}^{Tx}(x_{n,v})+G_{v}^{Rx}(x_{v,n})-L_{n}^{Tx}-L_{v}^{Rx}-L_{n,v}^{FS},$$

where *I_n_* (in dBW) is the received interference in the victim from the interfering ground station *n*; $P_{n}^{Tx}$ represents the transmitted power (in dBW) of each *n*-th interfering; $G_{n}^{Tx}(x_{n,v})$ stands for the off bore-sight gain (in dBi) of the *n*-th transmitting interfering station to the victim antenna; $G_{v}^{Rx}(x_{v,n})$ denotes the off bore-sight gain (in dBi) of the receiving victim antenna from the *n* interfering station; $L_{n,v}^{FS}$ indicates the free-space path loss, in decibels, between each *n* interfering station and the victim; and $L_{n}^{Tx}$, $L_{v}^{Rx}$ are the equipment losses of station *n* and the victim, respectively. The free-space path loss is given by the following relationship:

(13) $$L_{n,v}^{FS}=20\log_{10}(d_{n})+20\log_{10}(f_{n})-27.55,$$

where *f_n_* is the frequency (in MHz) of each *n* interferer, and *d_n_* is the ground distance, in meters, between the *n*-th station and the victim, as shown in Figure 1. Every CIR received from a single *n*-th transmitter (*n* = 1) at the interfered terrestrial station must satisfy the following norm:

(14) $$\frac{C_{sat}}{I_{n}}\geq {\left(\frac{C}{I}\right)}_{\min},$$

where *C_sat_* stands for the received carrier power at the interfered station, given by Equation (1), and *I_n_* denotes the received interference at the victim from the *n*-th transmitting station. These values are in clear numbers. Finally, (*C*/*I*)_min_ is the minimum acceptable value at the receiver (victim), from which it is determined if interference occurs or not. This value is usually mandated by the provider. In the case of multiple interferers (*n* > 1), the total CIR, concurrently received by *n* stations, is calculated according to

(15) $${\left(\frac{C}{I}\right)}_{total}={\left[\sum_{n=1}^{N}{\left(\frac{C_{sat}}{I_{n}}\right)}^{-1}\right]}^{-1}\geq {\left(\frac{C}{I}\right)}_{\min},$$

where (*C*/*I*)*_total_* denotes the total CIR, *I_n_* stands for the interference received from the *n*-th station that is given by Equation (11), and *N* indicates the total number of the interfering stations [10,18]. All the parameters in Equation (15) are in clear numbers.

The dominant atmospheric phenomenon affecting satellite and terrestrial communications at frequencies above 10 GHz is induced attenuation due to rainfall rate [6]. It is also important since it exhibits space–time variations. The whole interference situation is further deteriorated due to differential rain attenuation between the wanted and the interfering path [5,6,7]. Therefore, to incorporate this effect, we consider a margin that reduces the level of CIR that may occur under rainy conditions. This margin depends on the frequency, the separation angle, and the exceedance probability [5,7]. For the rest of this paper, a constant value of 5 dB is assumed, making the whole analysis more straightforward.

**Figure 4:**
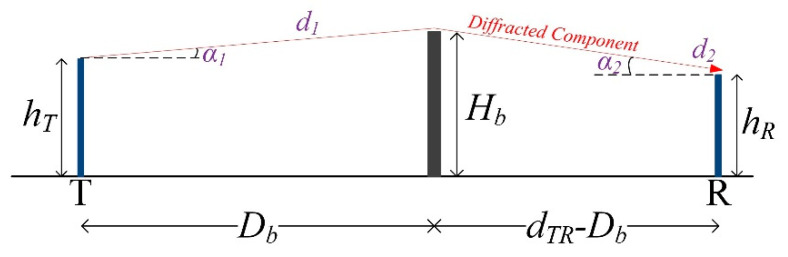
Basic principle of the knife-edge diffraction theory.

It is worth remarking that the total interference calculation is based on the worst-case assumption that no man-made constructions intervene in the direct path of interference between the two stations. In reality, there are obstructions (buildings, trees, or other ground stations) that may intervene between one transmitting interferer and a receiving victim, providing the required line-of-sight (LOS) blockage. Therefore, the interference values given by Equation (12) are expected to be further attenuated due to the aforementioned obstructions, thus increasing the achievable CIR. The penetration loss, especially at the Ka-band, is extremely high, so there is no chance of interference from the direct path since non-line-of-sight (NLOS) conditions occur, attenuating the signal severely. However, it is critical to examine the case of interference originating from a diffracted component. In the following, the analysis is based on the basic principle of knife-edge diffraction theory [19], as shown Figure 4.

The diffracted component from an obstacle (or barrier) between a transmitter (T) and a receiver (R) is attenuated due to the presence of the “knife-edge”, and the diffraction loss can be calculated from [19]

(16) $$L_{diff}=6.9+20\log_{10}\left(\sqrt{{\left(u-0.1\right)}^{2}+1}+u-0.1\right),$$

where *L_diff_*, denotes the diffraction loss, in decibels, and *u* is the diffraction parameter that is given by

(17) $$u=\theta \sqrt{\frac{2d_{1}d_{2}}{\lambda (d_{1}+d_{2})}},$$

where *λ* is the signal wavelength, in meters; *d_1_* is the distance from the transmitter to the obstacle; and *d_2_* is the distance from the obstacle to the receiver, both defined in meters, as shown in Figure 4. Furthermore *θ*, in radians, is given by

(18) $$\theta =a_{1}+a_{2},$$

and

(19) $$\begin{array}{l} a_{1}=\tan^{-1}\left(\frac{H_{b}-h_{T}}{d_{1}}\right)\\ a_{2}=\tan^{-1}\left(\frac{H_{b}-h_{R}}{d_{2}}\right) \end{array}$$

(20) $$\begin{array}{l} d_{1}=\sqrt{{\left(H_{b}-h_{T}\right)}^{2}+D_{b}^{2}}\\ d_{2}=\sqrt{{\left(H_{b}-h_{R}\right)}^{2}+{\left(d_{TR}-D_{b}\right)}^{2}} \end{array}$$

where *H_b_* represents the obstacle height (or the intervening buildings), in meters, and *h_T_*, *h_R_* are the total heights, in meters of the transmitter and receiver, respectively. Furthermore, *D_b_* denotes the distance, in meters, of the obstacle from the transmitter, and *d_TR_* (in m) is the total separation between transmitter and receiver, as shown in Figure 4 (*d_TR_* = *d_n_*). Based on the above, one can determine the loss of the diffracted component using simple geometric calculations. The diffraction loss is calculated for each one of the *n* transmitting stations to the victim, and the result is subtracted from *I_n_*, given by Equation (12). Finally, it is important to distinguish the height of the aperture center above the ground, *h_e_* (which is the antenna center height above the ground, in meters), and the total height, *h_T,R_*, in meters, which includes the antenna dish. The total height is given by

(21) $$h_{T,R}=h_{e}+\frac{d_{ant}}{2}\cos\theta ,$$

where *d_ant_* is the antenna dish diameter, in meters, and *θ* is the antenna’s elevation angle in degrees [20]. Finally, it should be pointed out that the lowest value of diffraction loss is yielded when the barrier is approximately in the middle of the path between T and R (*D_b_* = *d_TR_*/2), whereas the maximum loss is obtained when it is located close to T or R, respectively [19]. The latter case is usually met when a building intervenes between the terminals. Therefore, a barrier can be regarded as the building facade and its corresponding height, which is closer to T or R. The final calculation procedure of CIR, between multiple interferers and a single victim, is described by Algorithm 1.

Finally, it should be pointed out that the proposed interference calculation methodology can be sufficiently applied in far-field scenarios as well, with the appropriate modifications. Algorithm 1 can be employed as is, changing only the equations for the antenna gain approximation. Therefore, in the case of far-field interference evaluation, Equation (9) will be employed in the methodology instead of Equation (11). Moreover, these are described in ITU-R SF-Series and SM-Series [21,22]. In these scenarios, the joint propagation phenomena should also be considered as an aggravation of the clear-sky interference [23,24].
**Algorithm 1.** Calculation of CIR between multiple interferers and a single victim.**Input:** (*φ_0_*, *λ_0_*), *θ_v_*, *ϕ_v_*, *EIRP_sat_*, *G_Rx,max_*, $L_{v}^{Rx}$, *f_sat_*. **Step 1:** calculate *d_sat_* using Equation (3) **Step 2:**
*if G_R-max_* = none, *then*   **Input:** *d_ant_*, *k*   **Step 1:** calculate *G_Rx,max_* using Equation (2)   **Step 2:** calculate *C_sat_* using Equation (1)  *else*   **Step 1:** calculate *C_sat_* using Equation (1)  *end* **Input:** N (number of interfering stations) **for**
*n* = 1 **to**
*N* (interferers)   **Input:** (*φ_n_*, *λ_n_*), *θ_e,n_*, *ϕ_n_*, $P_{n}^{Tx}$, $L_{n}^{Tx}$, *f_n_*. Select condition (LOS/NLOS) between each interferer and victim.   **Step 1:** calculate *d_n_* using Equation (4)   **Step 2:** calculate *θ_b,n_* using Equation (5)   **Step 3:** calculate *x_n,v_* using Equation (7)   **Step 4:** calculate $G_{n}^{Tx}(x_{n,v})$ using Equation (11)   **Step 7:** calculate *x_v,n_* using Equation (8)   **Step 8:** calculate $G_{v}^{Rx}(x_{v,n})$ using Equation (11)   **Step 9:** calculate $L_{n,v}^{FS}$ using Equation (13)   **Step 10:** calculate *I_n_* using Equation (12)   **Step 11:** *if* LOS conditions are selected, *then*      **Step 1:** calculate *C_sat_*/*I_n_* using Equation (14)      **Step 2:** *C_sat_*/*I_n_* = *C_sat_*/*I_n–_*5 (subtract 5 dB for differential rain fade margin)     *else*      **Input:** *h_e_* (antennas’ center above ground), *H_b_, D_b_* (select the distance of the barrier closest to T or R)      **Step 1:** calculate *h_T,R_* using Equation (21)      **Step 2:** calculate *L_diff_* using Equations (16)–(20)      **Step 3:**
*I_n_* = *I_n–_L_diff_*      **Step 4:** calculate *C_sat_*/*I_n_* using Equation (14)      **Step 5:** *C_sat_*/*I_n_* = *C_sat_*/*I_n–_*5 (subtract 5 dB for differential rain fade margin)     *end*      **Output:** *C_sat_*/*I_n_* **end** **Input:** (*C*/*I*)_min_ **Step 3:** calculate (*C*/*I*)*_total_* using Equation (15), the rain fade margin is already considered. **Step 4:** *if* (*C*/*I*)*_total_* > (*C*/*I*)_min_ *then*   **Output:** “No interference found”  *else*   **Output:** “Possible interference, please apply mitigation countermeasures”  *end* **Output:** (*C*/*I*)*_total_*

### 2.2. Numerical Results and Discussion

**Figure 5:**
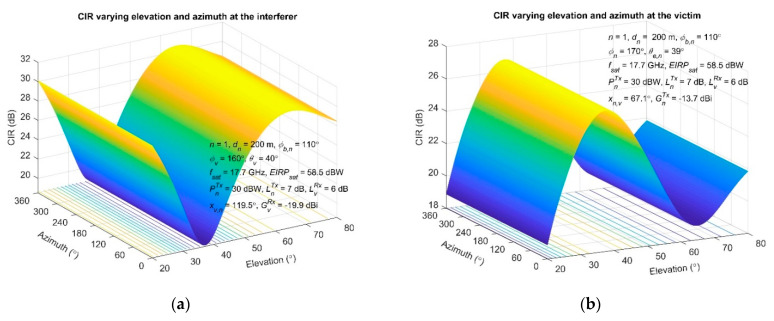
Achieved CIR at the victim side, varying the elevation and azimuth: (**a**) at the interfering station; (**b**) at the victim station.

In the following, different scenarios are examined, adjusting the geometrical and/or technical parameters of the terminals so as to assess their influence on the interference outcome. The CIR variation, as a function of both the azimuth and elevation angles of the interferer and victim, is presented in Figure 5a,b, respectively. A single interferer is considered (*n* = 1) when applying Algorithm 1, and LOS condition as the worst-case scenario. In both cases, the CIR is maintained constant, with the azimuth variation having negligible effect. On the other hand, the adjustment of the elevation angle introduces significant variations on the CIR, being on the order of 18.6–30.6 dB and 21.5–30 dB at the interferer and victim side, respectively. According to the results, the CIR variation magnitude at the interferer side is greater, reaching almost 11.9 dB, whereas CIR minimizes as the interferers’ elevation angle (37°) becomes comparable with the victim’s (*θ_v_* = 40°), as one can observe in Figure 5a. The maximum CIR is achieved for an elevation angle of 65°. Similarly, at the victim side, the CIR maximizes as the elevation angle of the victim station (38°) approaches that of the interferer (*θ_e,n_* = 39°), as shown in Figure 5b. Additionally, CIR presents its minimum for an elevation angle of 65°. The general trend is that the local minimum of the CIR at the interferer side is reflected as a local maximum on the victim location, and vice versa, as the plots indicate in Figure 5. (The difference between these two points is about 28°.) It is clearly attributed to the selected geometry, on the basis of Equations (7) and (8) and it is subjected to changes demonstrating a reversed tendency if *ϕ_b,n_* > 180°.

**Figure 6:**
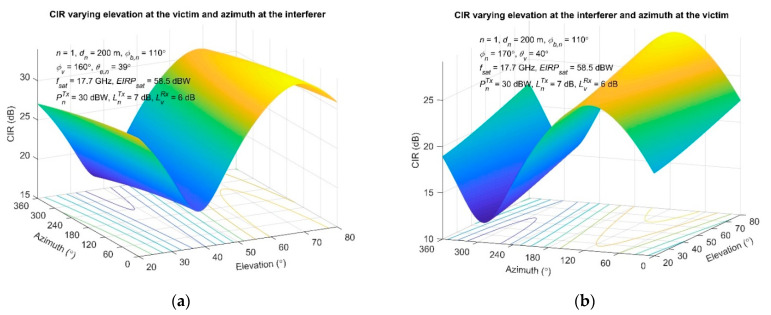
Achieved CIR at the victim side varying the parameters: (**a**) elevation at the victim and azimuth at the interfering station; (**b**) elevation at the interfering station and azimuth at the victim station.

Furthermore, a combined angle modification scenario is examined in Figure 6, where the CIR is assessed varying the elevation angle at the victim and the azimuth angle at the interferer side, as well as the opposite case. A single interferer is assumed (*n* = 1) with LOS propagation condition between the terminals.

From Figure 6a, it is clear that the azimuth variation at the interferer station also has an impact on the obtained CIR at the victim side. More specifically, CIR drops about 3 dB as the azimuth angle increases, and this difference between the maximum and minimum points occurs for the entire range of the elevation angle. On the other hand, CIR exhibits significant fluctuations as the elevation angle changes at the victim, taking values between 18.1 and 32.5 dB, on average. The minimum CIR is presented when the victim’s elevation angle (38°) approximates the interferer’s elevation angle (*θ_e,n_* = 39°), as shown in Figure 6a, whereas the CIR maxima is obtained when the victim station has an elevation angle of 68°. This CIR tendency versus elevation angle (maximum/minimum points), shown in Figure 6a, stands for *ϕ_b,n_* < 180° and exhibits a reversed behavior as *ϕ_b,n_* > 180° (the maximum point becomes minimum, and the opposite). Furthermore, four different cases can be distinguished regarding the CIR variations with both elevation and azimuth in conjunction with the bearing angle:

- if *ϕ_b,n_* < 90°, then the CIR decreases proportionally to the azimuth angle (about 2.2 dB), retaining the same trend with Figure 6a, regarding the maximum/minimum CIR points versus the elevation angle,
- if 90° < *ϕ_b,n_* < 180°, the CIR drops as the azimuth angle increases (about 3 dB, as already shown in Figure 6a),
- if 180° < *ϕ_b,n_* < 270°, the CIR reduces as the azimuth angle escalates, having a reduction of about 1.5 dB, whereas the CIR maximum/minimum points versus elevation angle are now inverted, and finally,
- if *ϕ_b,n_* > 270°, then the CIR increases about 8.5 dB as the azimuth angle grows, and the CIR fluctuation versus elevation angle exhibits the trend opposite of the one shown in Figure 6a.

Additionally, there are obvious similarities in the two scenarios presented in Figure 5a and Figure 6a. The results imply that with changing the elevation angle, either at the interfering or at the victim station, the CIR demonstrates severe variations. It takes its lowest value at an angle comparable with the elevation angle of the interferer and victim, provided that these two are similar (*θ_e,n_* ≈ *θ_v_*).

Totally opposite results are obtained in Figure 6b, where the azimuth angle is modified in the victim and the elevation angle at the interfering terminal. The results show that, in this case, CIR variates significantly with the azimuth angle, presenting a different performance than the one shown in Figure 6a. The adjustment of the victim’s azimuth angle introduces CIR variations on the order of 12.1–25.7 dB and 15.6–29.1 dB, which depend on the interferers’ elevation angle. The variation magnitude is about 13.6 dB, on average. The maximum value of the CIR is obtained for an azimuth angle of 107°, a value that approaches the selected bearing angle (*ϕ_b,n_* = 110°). On the other hand, the CIR minimizes as the azimuth angle becomes 270°. The specific local maximum/minimum angle points remain stable, being independent of the elevation angle, as one can observe in Figure 6b. Furthermore, the elevation angle adjustment also produces a CIR deviation, which increases proportionally with a magnitude on the order of 3.4 dB. The bearing angle affects the tendency of the produced CIR, in combination with the azimuth and elevation angles, where the trend shown in Figure 6b is inverted if *ϕ_b,n_* > 180°. Therefore, four different cases can be categorized:

- if *ϕ_b,n_* < 90°, then the CIR decreases inversely, proportionally to the elevation angle (approximately 2.6 dB), retaining the same trend as with Figure 6b, regarding the maximum/minimum CIR points versus azimuth angle,
- if 90° < *ϕ_b,n_* < 180°, then the CIR escalates as the elevation angle increases (about 3.4 dB as already shown in Figure 6b),
- if 180° < *ϕ_b,n_* < 270°, the CIR maximum/minimum points versus azimuth angle are now inverted, and the CIR increases as the elevation angle grows (about 3.5 dB), and finally,
- if *ϕ_b,n_* > 270°, the CIR preserves the same reversed trend with the azimuth angle, and it drops as the elevation angle increases (approximately 3.6 dB).

**Figure 7:**
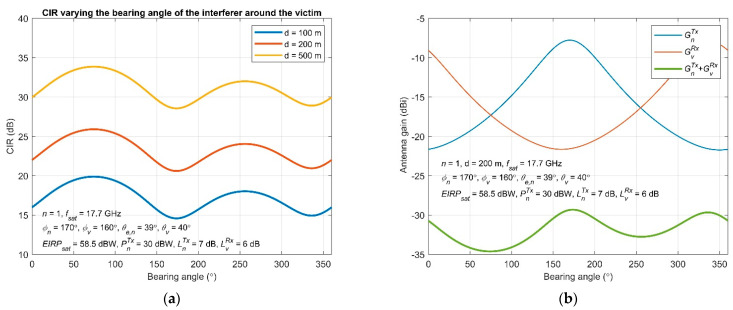
Achieved CIR by changing the location of the interfering station around the victim: (**a**) varying the bearing angle around the victim for diverse separation distance; (**b**) antenna gain variations with respect to the bearing angle.

The CIR variations as a function of the bearing angle, for different separation distances between the interferer and the victim, are depicted in Figure 7a. Due to the induced free-space loss, on the basis of Equation (13), the distance demonstrates a clear impact on the expected CIR, which decreases as the interferer and the victim come closer. If the distance is doubled (i.e., from 100 to 200 m), the CIR increases about 6 dB. The CIR variates significantly with the bearing angle as well, exhibiting maximum and minimum points due to the selected geometry. Therefore, the bearing angle directly affects the CIR on the victim if the interferer is placed at different locations around it. In the specific scenario, the CIR maximizes at 75° and 170°, whereas it takes its lowest value at 256° and 335°, respectively.

The CIR variations in Figure 7a are fully reflected by the obtained results shown in Figure 7b, where the antenna gain variability is expressed as a function of the bearing angle. The rest of the parameters are the same, and *d* = 200 m. The total antenna gains from the interferer and the victim stations, $G_{n}^{Tx}$ + $G_{v}^{Rx}$, shown in Figure 7b, corroborate the CIR trend presented in Figure 7a, and these two parameters (and in respect, the off bore-sight angles) regulate the interference level given by Equation (12). The local maximum (minimum) points of CIR are attributed to the (low) high aggregate gain of the antennas, shown in Figure 7b. Finally, the gains plotted independently for the victim and interferer indicate the gain tendency with respect to the off bore-sight angle since the gain reduces as this angle increases, according to Figure 3. The gain curves between the interferer and the victim also exhibit an opposite trend, where a smaller off bore-sight angle (and gain) at the interferer entails a larger angle (and gain) at the victim, and vice versa. Therefore, based on the above, the CIR maximizes when these off bore-sight angles become comparable as well as the corresponding gains.

**Figure 8:**
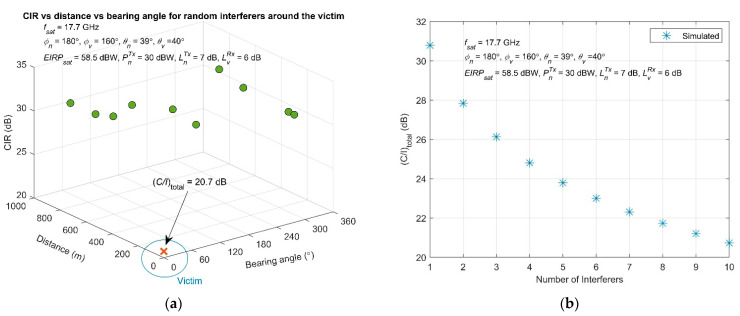
Examined scenario having multiple interferers around the victim: (**a**) achieved independent CIR for each interferer for random distance and bearing angle. The total CIR is also depicted; (**b**) obtained total CIR at the victim side versus the number of interfering stations.

The coexistence of multiple interfering stations and their impact on CIR is evaluated in Figure 8. More specifically, Figure 8a presents the CIR at the victim terminal for 10 different interferers around it, having a random distance and bearing angle. A clear LOS condition is also assumed. The combined interference, according to Equation (15), is also provided. Each interferer results in a CIR that varies from 28 dB up to 32, although the obtained composite interference drops down to 20.7 dB, simultaneously caused by the 10 stations. For the same parameters, Figure 8b shows the additive behavior of the number of interfering stations to the total CIR. The results demonstrate that the CIR decreases gradually in the presence of more interfering stations around the victim, varying from 30.8 dB for 1 interferer, down to 20.7 dB if 10 interferers exist simultaneously. 

Based on the above analysis and numerical results, it is evident that the three basic parameters that affect the achieved CIR on the receiver station (victim) are the bearing angle, the azimuth angle, and the elevation angle. Therefore, the selected geometry clearly accounts for the CIR variations producing different tendencies, thus minimizing and maximizing at specific angular locations of the interferer around the victim terminal.

## 3. Application Scenario

**Figure 9:**
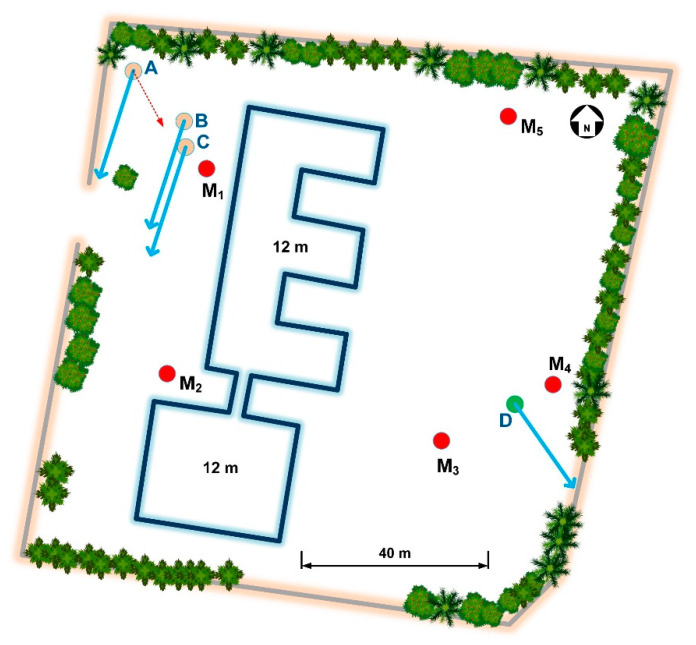
Ground plan of the teleport facilities where a real interference scenario is assessed.

In the following, a real interference scenario analysis is presented, which involves the installation of three new ground stations for FSS in a small teleport facility. The ground plan of the facility is shown in Figure 9, along with the satellite station locations. Their average altitude is 110 m above sea level. The scenario is the installation of three new terminals (A, B, and C, depicted in Figure 9) that transmit at the Ka-band for FSS. However, these may cause harmful interference to the feeder link of the BSS that is collocated in location D with spectrum sharing in the Ka-band, as well. The blue arrows indicate the azimuth orientation of the antennas with respect to the north. Between the new and the existing terminal, there is a 12 m in height building where the management services are hosted.

The detailed technical characteristics of the corresponding satellite stations are listed in Table 1. According to the parameters, the earth-to-space signal (uplink) that is transmitted by stations A, B, and C, may interfere with the space-to-earth signal (downlink) received by station D. It is worth remarking that the antenna gain for station D is already given, consequently Equation (2) is not applied in Algorithm 1.

Furthermore, *ϕ* and *θ* are the azimuth and elevation angles, and *L_t_* and *d_ant_* are the equipment losses and the dish diameter, respectively. The height, *h_e_*, is the dish center of the antenna above the ground. Finally, according to the provider, *EIRP_sat_* = 58.5 dBW for station D. Applying Algorithm 1 for *N* = 3, the obtained results for CIR, independently, as well as the total interference, are summarized in Table 2. It should be commented that the results are initially provided without calculating the diffraction loss, so as to examine the effect of the propagation condition in the absence and presence of the intervening building (LOS or NLOS condition). Finally, (*C*/*I*)_min_ = 25 dB, as requested by the provider. According to Table 2, there is no chance of interference if these three stations are to be installed. Even independently, the obtained CIR at station D is on the order of 53.6–56.8 dB, far above the requested threshold. Their cumulative interference results in a CIR of 50.6 dB, which overcomes the imposed limit. However, if there were no building between the stations (which occurs in many cases in congested teleport facilities), the achieved CIR, independently for the three interfering stations, is below the limit (20.6–23.5 dB). The achieved CIR for the total interference is 16.6 dB, which indicates the existence of harmful interference at station D, thus considering appropriate mitigation countermeasures. Therefore, the results in Table 2 imply that, in LOS conditions, an interference incidence is highly likely in the teleport facilities when spectrum sharing scenarios are selected at the Ka-band and the separation distance between the interfering and victim terminals is close (90–110 m).

Finally, the existence of a man-made barrier creates an effective shielding to the victim terminal, significantly reducing the interfering signals. If this barrier is close to a terminal, the induced diffraction loss is higher, as one can infer from Table 2, comparing the loss between stations B, C, and A.

## 4. Methodology Validation with Measurements

The algorithmic procedure presented in Section 3, which describes the interference calculation methodology, is validated through measurements carried out in the same facilities depicted in Figure 9. The scenario incorporates the interference assessment between the proposed methodology and the measured interference at various locations induced by an experimental terminal. The latter comprises a 2.4 m antenna and transmitted a modulated 1 MHz bandwidth signal between 17,816–17,821 MHz (carrier frequency at 17,819 MHz). The testing terminal is fixed at location A, pointing at the red, dashed arrow shown in Figure 9. The antenna’s elevation and azimuth orientation are 43.3° and 156.2°, respectively. The uplink earth station EIRP with 1 MHz modulation is 62.5 dBW, and the dish center of the antenna is at 2 m above the ground. The transmit power is 11.7 dBW. The received interfering signal is measured at five different locations around the facilities (M_1_–M_5_), as indicated in Figure 9. It should be pointed out that there is a LOS condition between locations M_1_, M_2_, and M_5_, and the experimental terminal, whereas positions M_3_ and M_4_ exhibit an NLOS condition. The measurements are conducted exploiting a spectrum analyzer, an omnidirectional antenna, and a 2 m low-loss cable. The receiving antenna has 3 dB beamwidths of 360° and 45° in the azimuth and elevation planes, respectively. It is fixed at 2 m above the ground on a wooden tripod. The antenna has a gain of 5.8 dBi, whereas the insertion loss of the cable is 3.4 dB at the selected measurement frequency.

The measurement procedure is based on specific methodology, as described in [25] and complies with the ISO/IEC 17025:2017 standard [26]. The utilized equipment is calibrated according to the ISO/IEC 17025:2017 standard as well. The expanded measurement uncertainty (*k* = 1.96) is 4.1 dB, for a 95% confidence interval. It is calculated according to [27] and incorporates Type B uncertainty, as well as the operator proximity uncertainty (1.2 dB for a 2-m cable) and the random reflection uncertainty (0.5 dB) that accounted for the presence of cars, employees, etc. The last two uncertainty factors do not depend on frequency and are determined in [28].

**Figure 10:**
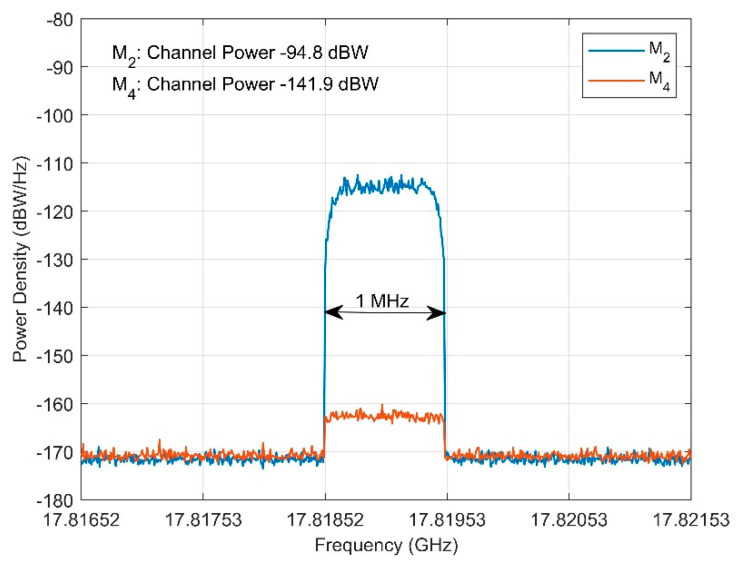
Measured power density for locations M_2_ and M_4_. The total channel power expressed across the 1-MHz transmitted bandwidth is also indicated.

During the measurements, the spectrum analyzer is automatically controlled through a GPIB interface and recorded, in each data set, 601 power density samples (in dBm/Hz) as a function of frequency. These samples incorporate the antenna gain and the cable loss, so they must refer to the received power density at the input of the reception antenna, thus describing the incoming interference. Therefore, the measured interference, expressed in dBW/Hz, is calculated according to

(22) $$I_{meas}^{i}=P_{r}^{i}-G_{ant}+L_{cable},$$

where $P_{r}^{i}$ is the power density of the *i*-th sample (in dBW/Hz), recorded by the spectrum analyzer; *G_ant_* denotes the gain of the receiving antenna; and *L_cable_* indicates the cable loss. At each measurement location, datasets of 601 power samples between 17,816 and 17,821 MHz are collected, whereas the measured interference power (in dBW) received for the total transmitted bandwidth of 1 MHz is calculated according to

(23) $$I_{meas}=10\log_{10}\left(\sum_{BW=1\;\mathrm{MHz}}I^{i}(f_{i})\right),$$

where *I_i_* is interference power sample (in Watts) at the specific frequency point *i*, and BW is the total summation bandwidth (1 MHz). The numerical results for each location are summarized in Table 3, whereas indicative measurement recording for locations M_2_ (LOS) and M_4_ (NLOS), are presented in Figure 10.

The measurement results are compared with the interference prediction obtained from the introduced methodology. In this case, the interference is considered and calculated at the input of the reception antenna. Therefore, Equation (12) and Algorithm 1 are modified accordingly

(24) $$I_{pred}=P_{Tx}+G_{Tx}(x)-L_{FS}\underset{\mathrm{NLOS}}{\underset{︸}{-L_{diff}}},$$

where *P_Tx_* stands for the transmitted power of the experimental station (in dBW); *G_Tx_*(*x*) denotes the off bore-sight gain (in dBi); *x* is the off bore-sight angle in degrees between the transmitting terminal and the measurement location; and *L_FS_* is the free-space loss, in decibels, given by Equation (13). The diffraction loss, *L_diff_*, in decibels, is taken into account only in NLOS locations and is calculated accordingly in Algorithm 1. The gains and losses of the receiving antenna are considered in Equation (22). Applying Algorithm 1, the predicted interference levels are listed in Table 3, along with the rest of the involved parameters. The interference level between the measurements and the proposed methodology is assessed and compared at the input of the reception antenna. As one can observe, the predicted theoretical values approximate the measured levels, and the demonstrated deviation between them falls within the uncertainty error (4.1 dB) of the measurement setup. In this context, the measurement results validate that the proposed methodology can predict with exceptional accuracy the ground-path interference between ground stations.

## 5. Interference Mitigation Techniques

As presented in Section 3, interference events are highly likely when there is a LOS condition between the interferer and the victim, especially at close distances in teleport facilities. In this case, application of specific mitigation countermeasures is vital so as to eliminate the chance of interference.

Frequency reallocation of the transmitting or receiving ground station bands is one option, where a proper selection of the frequency carriers could suppress interference. However, this selection is more conveniently applied in new station installations and not in existing ones. Nevertheless, this alternative may undergo difficulties in its implementation due to spectrum congestion.

Another mitigation option is to reallocate the interferer of the victim terminal. In this case, a careful establishment of the station is important, taking advantage of the surroundings and terrain so as to shield the terminal of interest, providing sufficient protection. More specifically, the satellite provider could re-install a terminal behind a building or other man-made construction so as to block the interfering signal. This can succeed even if the station is placed behind an existing ground station, thus blocking the LOS path. The aforementioned technique can be implemented easily in new installations. However, it is a very cumbersome and costly task to reallocate existing stations.

The most common technique, which is easy to apply with affordable cost, is site shielding. In this case, special barriers are constructed and placed in front of the endangered terminals, thus providing, on the one hand a direct block of the harmful signal, and on the other hand, sufficient diffraction loss. The purpose is to select a barrier at a specific distance from the protecting terminal, having a proper height, length, and material type. Conventionally, the satellite providers prefer to put barriers at the victim side so as to maximize the shielding and effectively protect the terminal.

Furthermore, the barrier has to provide adequate LOS blockage (attenuation) as well as a sufficient diffraction loss, in order to effectively protect the selected station. The best solution for the material type would be a mesh grid (made from either steel or aluminum). The mesh shield will have attenuation versus frequency response that will be a function of the material openings. The characteristics of an open construction shield will approach those of a solid shield, as the materials openings (holes) are much smaller than the wavelength of the signal being shielded (smaller than 17 mm for the Ka-band). Open construction is generally cheaper and easier to work with, providing less wind resistance than solid shields. An approximate expression for the transmission loss of a square-grid wire mesh (wire diameter much smaller than the wavelength) is given by

(25) $$L_{LOS}=20\log_{10}\left(\frac{\lambda }{2g}\right),$$

where *g* stands for the side length of the square, and *λ* is the wavelength of the signal [20]. For example, if a mesh barrier with *g* = 20 mm is selected, then for *λ* = 17 mm, *L_LOS_* = −7.4 dB, providing an adequate attenuation of the LOS signal. In the case of a solid aluminum or steel barrier, then *g* ⇾ 0, and *L_LOS_* ⇾∞, acting as a perfect reflector, eliminating the direct (LOS) interference from the interfering station. As proposed, materials could be exploited:

- *Aluminum*–Using aluminum for RF shielding requires particular attention to its oxidation characteristics and its galvanized corrosion potential. Aluminum should never be placed in direct contact with cement or concrete. Being non-ferrous, aluminum also exhibits reduced low-frequency magnetic field shielding.
- *Steel*–Steel in its various forms (e.g., galvanized, annealed, un-annealed, hot-rolled, cold-rolled, etc.), is the third metal commonly used for RF shielding. Steel used for RF shielding is typically protected from the environment for the duration of its use. Iron oxide (rust) will form to some extent and must be removed.

The barrier length *L_b_*, in meters, can be calculated from the following expression:

(26) $$L_{b}=1.5\cdot \max\{d_{ant}^{T},d_{ant}^{R}\},$$

where $d_{ant}^{T}$ and $d_{ant}^{R}$ are the antenna dish diameters of interfering and victim station, respectively. In this context, the barrier length depends directly on the dish diameter and, according to Equation (26), should be selected 1.5 times greater than the maximum antenna dish. For example, considering antennas having diameters of 7.2 m and 4.8 m at the victim and the interferer, respectively, the barrier length should be selected greater than 10.8 m. The minimum distance of the barrier *D_b_*, in meters, from the station under protection, can be calculated for a given barrier height, Hb, in meters, according to the following expression:

(27) $$d_{b}=\frac{H_{b}-h_{e}}{\tan\theta_{v}}+\frac{1.5d_{ant}}{2\sin\theta_{v}},$$

where *h_e_* denotes the height of the aperture center above the ground, in meters; *d_ant_* indicates the antenna dish diameter, in meters; and *θ_v_* stands for the victim’s antenna elevation angle in degrees [20]. Therefore, properly selecting the barrier height, usually higher than the total height given by Equation (21), one can determine the optimum distance, *D_b_*. Considering, for example, *h_e_* = 6.2 m and *d_ant_* = 7.2 m, a total height of 8.8 m is obtained from Equation (21). In this case, the barrier should be at least higher than 9 m. Selecting *θ_v_* = 44.7°, and *H_b_* = 10 m, then according to Equation (27), the minimum distance is obtained, 11.5 m, so the barrier should not be established closer than this value. A final step is to calculate the induced diffraction loss from the barrier at the specific location in order to eliminate any chance of interfering signals that originate from the diffraction mechanism. The applied procedure can be found in Section 3.

Finally, a more advanced technique for interference mitigation is beamforming although it is a difficult and costly alternative for existing terminals. The beamformer operates as a spatial filter at the output of an antenna array in order to form a desired beam pattern [29]. The signals impinging at the various elements of the antenna are combined, forming a single output. The beamforming operation can be discriminated in two parts, such as synchronization and weighted sum. The former part assures the coherent transmission or reception of the signals from the different antenna elements, and the latter technique determines and controls the steering direction towards the intended receiver as well as controlling the beamwidth of the main lobe and the characteristics of the sidelobes of the antenna. Beamforming techniques can be implemented either in the interferer or in the victim side. It is worth remarking that the application of a beamforming technique necessitates a substantial upgrade in the existing FSS or BSS terminals. Therefore, a major improvement is required, as a terminal equipped with multiple antennas is necessary to create a convenient beam pattern. The main goal is to create beam patterns with null points at the direction of the victim (or interferer) for the specific off bore-sight angles.

## 6. Conclusions

This article presented a unified, straightforward and detailed methodology in a tutorial style for the ground-path interference calculation between FSS and BSS terminals that coexist in teleport facilities and share the same spectrum in the Ka-band. The proposed procedure took into consideration the near field of the ground stations, proposing a modification for the calculation of the off bore-sight gains of both terminals. The proposed methodology can also be applied to interference scenarios in other frequencies where the assumptions are still valid. The methodology was applied in a real application scenario, and various numerical results were provided. The numerical analysis showed that the geometrical parameters of the ground terminals played a vital role in the obtained CIR. The bearing angle of an interfering station around the victim, as well as the azimuth and elevation angles of both terminals, produced significant angular variations of the CIR, exhibiting local minimum and maximum values that depended directly on the above-mentioned parameters.

Furthermore, the adjustment of the azimuth and elevation angles, either at the interfering or at victim terminals, showed that the azimuth angle does not produce variations on CIR. On the other hand, the modification of the elevation angle introduces significant variations on the CIR, being on the order of 18.6–30.6 dB and 21.5–30 dB at the interferer and victim side, respectively. The CIR variation magnitude at the interferer side is greater, reaching almost 11.9 dB. Additionally, when varying the elevation at the victim and the azimuth at the interfering station, the CIR drops by about 3 dB as the azimuth angle increases. However, the CIR exhibits significant fluctuations as the elevation angle changes at the victim, taking values between 18.1 and 32.5 dB, on average. Totally opposite results are obtained when the azimuth angle is modified at the victim and the elevation angle at the interfering terminals. The adjustment of the victim’s azimuth angle introduces CIR variations on the order of 12.1–25.7, dB and 15.6–29.1 dB that depend on the interferers’ elevation angle. The variation magnitude is about 13.6 dB, on average. The above-mentioned variations on CIR and the local maxima/minima tendency is found to be affected by the bearing angle, *ϕ_b,n_*, in conjunction with the azimuth and elevation angles.

The distance between the terminals demonstrates a clear impact on the induced CIR. If the distance is doubled, the CIR increases by about 6 dB. Moreover, the coexistence of multiple interfering stations results in CIR variations on the range of 28–32 dB. However, the composite interference caused by those 10 stations reduces down to 20.7 dB, showing an additive trend of the number of interfering stations to the total CIR. Finally, the results showed that the CIR decreases gradually in the presence of more interfering stations, varying from 30.8 dB for 1 interferer, down to 20.7 dB for 10 coexisting interfering stations.

From the measurements scenario, it has been shown as obvious that small uncertainties in the input parameters do not affect the interference results. The bias to the results is incremental.

Furthermore, man-made constructions, such as buildings, provide significant shielding if these are located amidst the ground terminals, increasing the diffraction loss and minimizing interference due to the induced NLOS propagation condition. This shielding may exceed 30 dB, substantially improving the CIR. On the other hand, the application of the theoretical methodology in LOS scenarios showed that there is an increased chance of interference between the examined ground terminals. Finally, measurements were carried out in a small teleport facility so as to support and validate the theoretical procedure. The obtained results of the calculated interference and the measured values show very good approximation. On this basis, the proposed methodology can be applied by satellite system engineers or regulatory authorities so as to predict accurately the ground-path interference between the involved terminals.

As future work, it can be considered to incorporate interference from adjacent radar systems operating above 10 GHz and also include adaptive power control. Moreover, statistical distributions of the propagation phenomena (rain-scatter interference and differential rain attenuation) can be included and provide C/I distributions for the calculation of the interference margins.

## Figures and Tables

**Table 1:** Technical Parameters of the Ground Stations for the Interference Assessment.

Parameter	Ground Station			
	A	B	C	D
*f_Tx_*	17.755 GHz	17.745 GHz	17.735 GHz	27.945 GHz
*f_Rx_*	11.833 GHz	11.928 GHz	11.814 GHz	17.740 GHz
*P_Tx_*	30 dBW	30 dBW	30 dBW	22 dBW
*G_Tx_*	60.0 dBi	57.0 dBi	57.0 dBi	66.5 dBi
*G_Rx_*	57.0 dBi	53.0 dBi	53.0 dBi	66.3 dBi
*L_t_*	8.5 dB	7.0 dB	7.0 dB	3.5 dB
*ϕ*	197.4°	197.4°	197.4°	144.6°
*θ*	44.7°	44.6°	44.6°	39.6°
*d_ant_*	7.2 m	4.8 m	4.8 m	9.2 m
*h_e_*	6.2 m	4.8 m	4.8 m	5.8 m

The location coordinates of the stations are not provided upon request of the administration.

**Table 2:** Interference Results from Stations A, B, and C, to Station D with and without Diffraction Loss.

Parameter	A → D	B → D	C → D
*n*	1	2	3
*d_n_*	106.59 m	90.42 m	87.45 m
*ϕ_b,n_*	131.55°	130.90°	127.95°
$G_{n}^{Tx}(x_{n,v})$	−14.60 dBi	−14.66 dBi	−14.95 dBi
$G_{v}^{Rx}(x_{v,n})$	−21.55 dBi	−21.53 dBi	−21.46 dBi
*L_n,v_*	97.96 dB	96.54 dB	96.25 dB
*I_n_*	−113.81 dBW	−110.93 dBW	−110.86 dBW
*C_sat_*	−85.31 dBW		
*C_sat_*/*I_n_*	23.50 dB	20.62 dB	20.55 dB
(*C*/*I*)*_total_*	16.58 dB		
*D_b_*	28.65 m	15.05 m	14.07 m
*L_diff_*	30.07 dB	36.04 dB	36.26 dB
*I_n_*	−143.88 dBW	−146.97 dBW	−147.12 dBW
*C_sat_*/*I_n_*	53.56 dB	56.66 dB	56.81 dB
(*C*/*I*)*_total_*	50.63 dB		
*(C/I)_min_*	25 dB		

**Table 3:** Comparison between Measured and Predicted Interference Levels.

Measurement Location	M_1_	M_2_	M_3_	M_4_	M_5_
*d_n_*	25.7 m	64.6 m	101.1 m	110.2 m	97.48 m
*ϕ_b,n_*	146.27°	173.96°	141.11°	126.62°	79.03°
*P_Tx_*	11.7 dBW				
$G_{n}^{Tx}(x_{n,v})$	−9.14 dBi	−9.60 dBi	−9.41 dBi	−10.63 dBi	−15.67 dBi
*L_n,v_*	85.66 dB	93.67dB	97.56 dB	98.31 dB	97.24 dB
*D_b_*	-	-	33.61 m	28.67 m	-
*L_diff_*	-	-	40.91 dB	41.00 dB	-
*I_pred_*	−83.1 dBW	−91.6 dBW	−136.2 dBW	−138.2 dBW	−102.2 dBW
*I_meas_*	−81.2 dBW	−94.8 dBW	−140.1 dBW	−141.9 dBW	−100.5 dBW

The interference levels are calculated across the total transmitted bandwidth of 1 MHz.

## Data Availability

Not applicable.
